# Frailty as a dynamic process in a diverse cohort of older persons with dialysis-dependent CKD

**DOI:** 10.3389/fneph.2023.1031338

**Published:** 2023-01-20

**Authors:** Nancy G. Kutner, Rebecca Zhang

**Affiliations:** ^1^ Department of Rehabilitation Medicine, Emory University School of Medicine, Atlanta, GA, USA; ^2^ Department of Biostatistics and Bioinformatics, Rollins School of Public Health, Emory University, Atlanta, GA, USA

**Keywords:** frailty, chronic kidney disease, hemodialysis, older persons, 4MS, resilience

## Abstract

This study examines frailty status evolution observed in a two-year follow-up of a cohort of older persons (age ≥65) with chronic kidney disease (CKD) undergoing maintenance hemodialysis (HD) treatment. Frailty, a geriatric syndrome that connotes a state of low physiologic reserve and vulnerability to stressors, is associated with increased risk for multiple adverse health outcomes in studies of persons with CKD as well as older persons in the general population. The Fried frailty index defines frailty as the presence of 3 or more of 5 indicators—recent unintentional weight loss, slowed gait speed, decreased muscle strength, self-reported exhaustion, and low physical activity. In the seminal work by Fried and colleagues, persons who were characterized by 1-2 of the Fried index criteria were termed “pre-frail” and considered at risk for subsequently becoming frail, potentially providing insight regarding intervention targets that might slow or prevent individuals’ transition from pre-frail to frail status. Other less frequently studied types of transitions may also be informative, including “recovery or reversion” (improvement) by people whose longitudinal assessments indicate movement from frailty to prefrailty or robust, or from prefrailty to robust. These status changes are also a potential source of insights relevant for prevention or remediation of frailty, but research focusing on the various ways that individuals may transition between frailty states over time remains limited, and no previous research has examined varying patterns of frailty status evolution in an older cohort of persons with dialysis-dependent CKD. In a study cohort of dialysis-dependent older persons, we characterized patterns of frailty status evolution by age, sex, race/ethnicity, and treatment vintage; by longitudinal profiles of non-sedentary behavior; and by self-report indicators relevant for dimensions emphasized in the Age-Friendly 4Ms Health System (What Matters, Mobility, Mentation). Our study suggests that strategies to promote resiliency among older persons with dialysis-dependent CKD can be informed not only by frailty status transition that indicates improvement over time but also by older adults’ maintenance of (stable) robust status over time, and we concur that inclusion of both frailty and resilience measures is needed in future longitudinal studies and clinical trials.

## Introduction

The concept of frailty is variably defined but is widely viewed as the loss of physiological reserve and increased vulnerability to stressors (1, 2). The well recognized frailty index developed by Fried et al. (1) defines frailty as the presence of 3 or more of 5 indicators—recent unintentional weight loss, slowed gait speed, decreased muscle strength, self-reported exhaustion, and decreased physical activity (**Table 1**). Individuals who are defined as frail by this index have increased risk for adverse health outcomes, including falls, functional decline and disability, long term care need, and mortality. Persons who are characterized by 1-2 indicators of the Fried index are considered “pre-frail” and at increased risk for becoming frail, constituting a study population of particular interest for identifying potential targets for interventions that might slow or prevent this transition. Applying the frailty phenotype to persons with dialysis-dependent CKD has provided “a novel approach for identifying disease burden and risks for adverse health outcomes” in this population (3).

**Table 1:** Fried frailty index: criteria and indicators^a^.

Frailty criteria	Indicators
Weight loss	Loss of >10 pounds in past 12 months, unintentional
Exhaustion	Response of “a moderate amount of the time (3-4 days)” or “most of the time” to either of two CES-D scale items: “I felt that everything I did was an effort”; “I could not get going” during the past week
Weakness	Maximal grip strength in kg using Jamar hand-held dynamometer. Lowest 20%, stratified by gender and BMI quartiles.
Slowness	Time in seconds to walk 15 feet at usual pace. Slowest 20%, stratified by gender and standing height.
Low physical activity level	Weighted score of kilocalories expended per week in physical activities “you have done in the past 2 weeks” reported on short version of Minnesota Leisure Time Activity questionnaire. Lowest 20% for each gender.
Frailty	Presence of 3 or more of the above criteria

BMI, body mass index; CES-D, Center for Epidemiologic Studies-Depression.

a Fried et al. (1).

In addition to individuals’ observed transition from pre-frail to frail status, changes in observed frailty status may indicate “recovery or reversion” (improvement) by persons whose longitudinal assessments indicate movement from frailty to prefrailty or even to robust, or movement from prefrailty to robust. These less frequently studied changes in frailty status represent a potential source of insights relevant for prevention or remediation of frailty (4). We suggest that these less studied patterns may also have implications for the concept of resilience in the context of aging, i.e. positive responses to high- or low-intensity stressors that facilitate return to equilibrium. Resilience is not the converse of frailty, nor does it reflect the absence of frailty; rather, frailty and physical resilience are closely linked. Longitudinal research may provide insight into this linkage when the human body is viewed as a complex system incorporating multiple physiological challenges to homeostatic balance (2).

**Figure 1:**
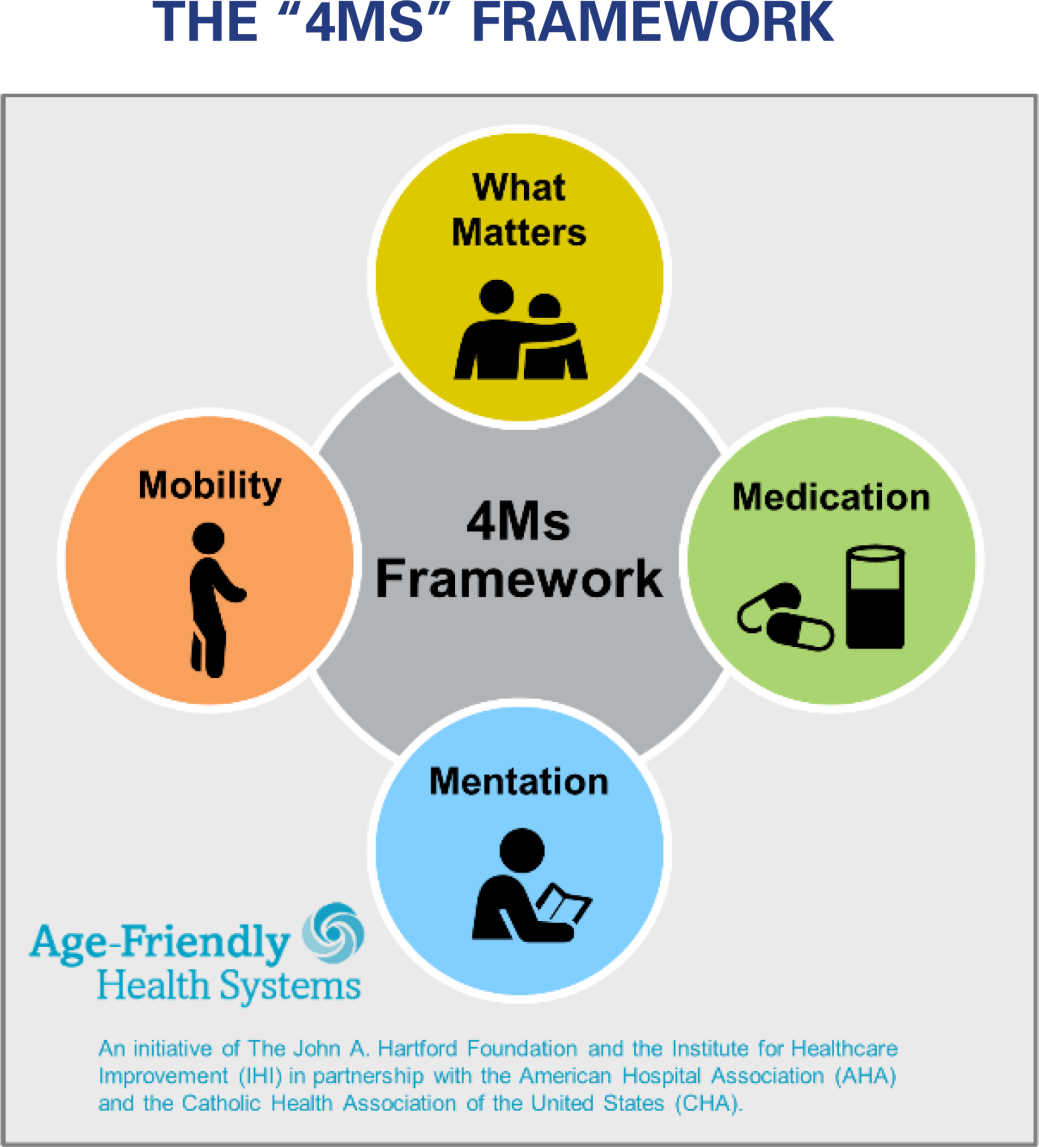
THE “4MS” FRAMEWORK. An Age-Friendly Health System is one in which every older adult’s care: Is guided by an essential set of evidence-based practices (the 4Ms); Causes no harms; and Is consistent with What Matters to the older adult and their family.

Frailty is considered a central organizing principle for effective care for older people, providing a predictive tool for outcomes that are valued by both patients and health systems (5). The Age-Friendly Health Systems (AFHS) model, which seeks to ensure that the “4Ms,” i.e. What Matters, Medication, Mentation, and Mobility, guide health care delivery for older adults (**Figure 1**), has been widely adopted, and continuing assessment of the model’s success in improving outcomes of older adults is considered essential (6). Our study considers the relevance of the 4Ms for outcomes observed in association with frailty status change among older persons with dialysis-dependent CKD.

In a cohort of older persons (age ≥65) with CKD receiving maintenance HD treatment, for whom frailty assessments were completed using the Fried protocol at baseline and two successive annual evaluations, the objectives of this study were (1) to categorize individuals by frailty status evolution pattern at their two-year follow-up, (2) to identify participant characteristics within frailty evolution categories, and (3) to examine across frailty evolution categories longitudinal profiles of participant non-sedentary behavior, and participant-reported indicators, that have relevance for the 4Ms Age-Friendly Health System priorities (**Figure 1**).

## Materials and methods

### Study population

The AAS [ACTIVE/ADIPOSE (A Cohort Study to Investigate the Value of Exercise in ESRD/Analyses Designed to Investigate the Paradox of Obesity and Survival in ESRD)] is the source of data for our analyses. The AAS is a multi-center longitudinal cohort study of prevalent kidney failure patients receiving in-center maintenance HD treatment that was jointly conducted in dialysis clinics in the Atlanta GA and San Francisco CA metropolitan areas. The study was coordinated by the United States Renal Data System (USRDS), which maintains a registry of all persons in the U.S. who receive treatment for kidney failure by dialysis or transplantation (www.usrds.org).

Data collection sites were 7 outpatient dialysis clinics in the Atlanta, Georgia metropolitan area and 7 outpatient dialysis clinics in the San Francisco Bay Area, California, at which a total of 771 patients were enrolled. Study inclusion criteria were: Adults (≥18 years), English- or Spanish-speaking, receiving HD for at least 3 months, and capable of providing informed consent. Exclusion criteria included current treatment by peritoneal dialysis or home HD, evidence of active malignancy, and imminent geographic relocation; vulnerable populations (pregnant women, prisoners, persons with significant mental illness) were also excluded. Among eligible patients undergoing HD at the study clinics during the 2-year enrollment period, 85% provided informed consent and were enrolled. Reasons most frequently given by those who declined to participate were that they were “not interested,” “too busy,” or “enrolled in another study.” Institutional review boards at Emory University and the University of California San Francisco approved the study, and all participants provided written informed consent.

**Figure 2:**
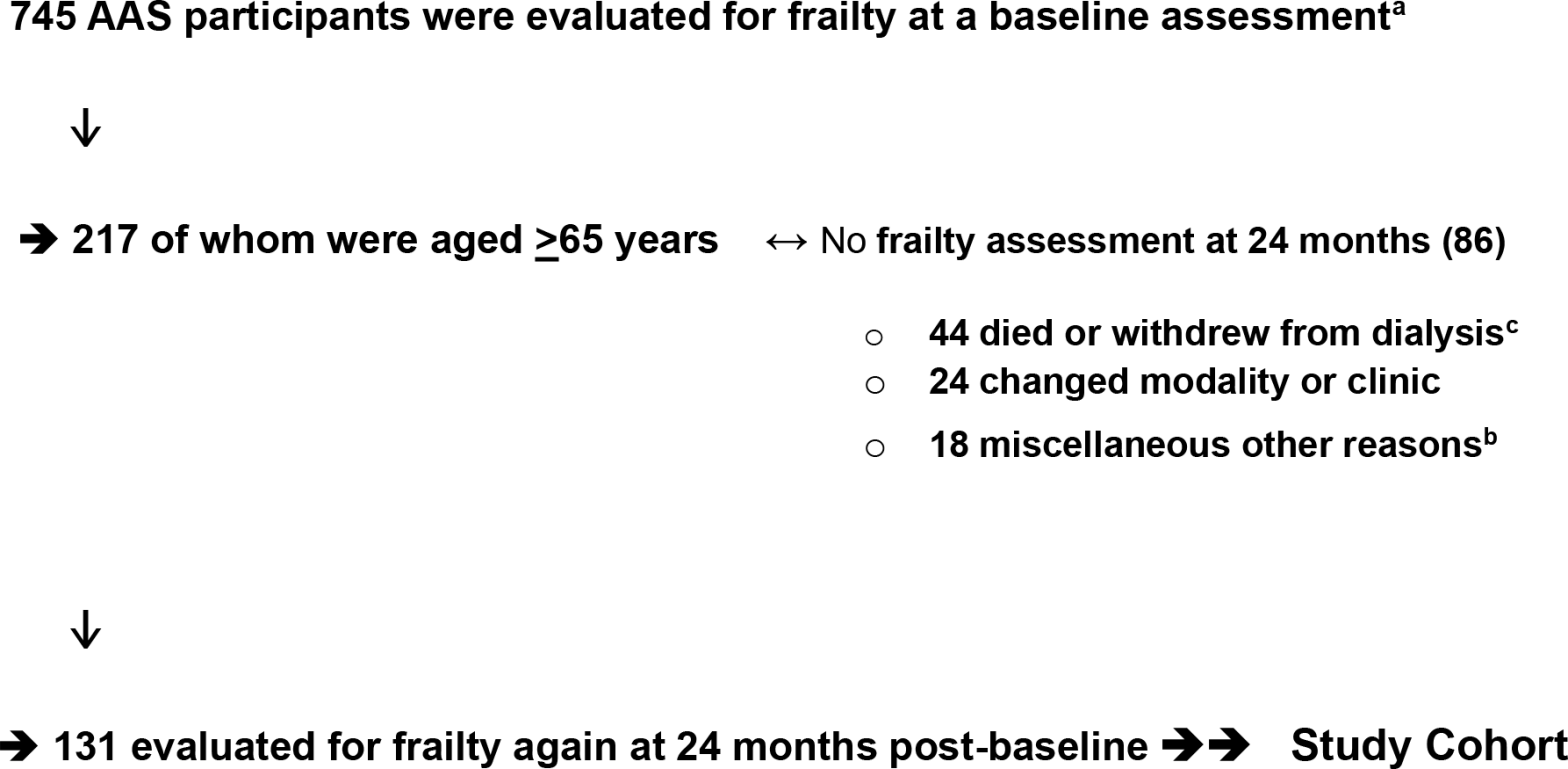
Derivation of Study Cohort: Older Persons with Dialysis-dependent CKD. 745 AAS participants were evaluated for frailty at a baseline assessment. a. ↓ → 217 of whom were aged ≥65 years ↔ No frailty assessment at 24 months (86). 44 died or withdrew from dialysisc. 24 changed modality or clinic. 18 miscellaneous other reasons. b. ↓ → 131 evaluated for frailty again at 24 months post-baseline →→ Study Cohort. AAS, ACTIVE-ADIPOSE Study a. Kutner et al. (7). b. Miscellaneous other reasons were observed for 18 participants: cognitive impairment; cancer; “too sick”; study withdrawal. c. Baseline frailty status of these 44 participants who died (n=41) or withdrew from dialysis (n=3): Robust=3; Pre-frail=23; Frail=18.

Study participants were enrolled and assessed at baseline 2009-2011, and two annual follow-up assessments were conducted for participants who remained in the study at these timepoints. Following the Fried et al. (1) methodology, a total of 745 AAS participants were evaluated for frailty at a baseline assessment, of whom 217 were aged ≥65. Of these 217 participants,131 also completed a frailty assessment at a 24-month follow-up, and these 131 participants comprise the study cohort for this paper. The primary reasons for older AAS participant attrition between baseline and the 24-month follow-up were death or withdrawal from dialysis (44 persons) and treatment modality change or transfer to a non-study clinic (24 persons) [**Figure 2**].

### Measures and data collection

Demographic and clinical variables, including age, sex, race/ethnicity, education, and length of time since start of treatment for kidney failure (vintage), were ascertained from patient report and USRDS Standard Analysis Files (SAFs). Educational status, race, and ethnicity were patient-reported; for the small number of participants who declined to specify their race or ethnicity, this information was taken from the USRDS Medical SAF. Older age, female sex, and lower educational status have been shown to be associated with higher likelihood of frailty among older adults in the general population (e.g. 1) and among older adult patients undergoing HD (8). Higher likelihood of frailty has also been observed for older persons in the general population who are African American (1), although black race was associated with lower odds for frailty in our baseline analysis of risk factors for frailty in the full (age ≥18) AAS cohort (7). Guo et al. (8) recently reported an association of longer vintage with frailty in a study of 204 persons age ≥60 receiving treatment for kidney failure in China.

#### Frailty assessment

Indicators specified by Fried et al. (1) were used to assess the 5 criteria of the frailty index: (1) weight loss in the past 12 months; (2) poor endurance and energy; (3) weakness, defined by grip strength; (4) slowness, defined by timed walk speed; and (5) low physical activity level (**Table 1**). Data sources included a brief interview with participants, medical record review, and performance measures of grip strength and walk speed. The maximal grip strength in kilograms was identified from 3 trials in both hands. Walk speed was the fastest time in seconds from 2 trials to walk 15 feet at the participant’s usual pace. The number of participants unable to walk was 3, two Hispanic white women ages 71 and 77, and one Asian man age 85; consistent with previous studies, participants unable to walk were classified in the slowest quintile for that indicator of the frailty index (9). Physical performance was assessed before HD on the midweek treatment day, and consistency of measurement procedures among study coordinators was monitored by the investigators. Study coordinators rescheduled the physical performance assessments as needed to accommodate participants who were tired, ill, or otherwise declined to complete the physical assessments on an originally scheduled day.

Consistent with the Fried et al. protocol (2001), assessment of physical activity level was based on responses to the Minnesota Leisure Time Activity Questionnaire, or LTPA (10), completed by study participants during an interview conducted by study coordinators. This instrument ascertains participation in various leisure time activities over the prior 2 weeks, e.g. walking, raking, gardening, dancing, swimming, hiking, biking, bowling. For each activity in which participants have engaged, information is also obtained about the frequency and duration of participation (time spent) in the activity, which is used to estimate the kilocalories (kcal) of energy expended per week in LTPA. Scoring of LTPA kcal/week to assess physical activity level followed the Cardiovascular Health Study algorithm, adjusting for gender (11), as used by Fried et al. (1).

#### Sedentary/non-sedentary profile of weekly energy expenditure

An individual who expends <500 kcal/week in LTPA over a 7-day period can be considered sedentary, and the cut point of 500 kcal/week therefore provides a metric for identifying participants with less active vs. more active behavior and lifestyle (12). Participants with <500 vs. ≥500 kcal/week estimated LTPA energy expenditure were classified as demonstrating a sedentary vs. non-sedentary profile at baseline and at 12- and 24-month follow-ups (13).

#### Self-reported indicators of 4Ms Age-friendly Health System priorities

- ***What Matters: SF-36 Vitality scale*** Energy/fatigue has been ranked by HD patients as their top priority outcome (14). The Vitality subscale of the SF-36 is the most frequently used measure of this outcome in research with persons receiving HD treatment (15). The SF-36 Vitality scale (score range 0–100) includes four items asking how much of the time during the previous 4 weeks the respondent felt worn-out, tired, and full of pep, and had a lot of energy (16). A Vitality scale score <55 provides a cut-point indicative of fatigue/low vitality (17); correspondingly, in the current study, participant-reported SF-36 Vitality scale scores ≥55 were considered indicative of perceived Vitality.
- ***Mobility: SF-36 Physical Function (PF) scale*** The SF-36 PF scale is a well-validated measure of self-reported physical function, with a strong association with poor walking speed (16). This measure (score range 0–100) includes ten items measuring whether health now limits physical function in moderate/vigorous activity; strength to lift, carry, stoop, bend, and stair climb; ability to walk various distances without difficulty, and self-care. A PF scale score <75 provides a cutpoint indicative of slowness/weakness (16); correspondingly, in the current study, an SF-36 PF scale score ≥75 provided an indicator of participants’ perceived mobility.
- ***Mentation: KDQOL Cognitive Function (CF) scale***. The Kidney Disease Quality of Life Cognitive Function (KDQOL-CF) scale (18) has been shown to be a valid instrument for estimating cognitive function in individuals with dialysis-dependent kidney failure, providing a valuable alternative in clinical care to time-consuming formal cognitive function screening and facilitating comparisons of cognitive function among different patient groups in epidemiologic studies (19). The KDQOL-CF scale (score range 0-100) includes three questions: During the past 4 weeks, did you react slowly to things that were said or done? Did you have difficulty concentrating or thinking? Did you become confused? The median scale score was 73 (interquartile range 60–87) for persons with ESRD in a validation study of the KDQOL-CF (19). Correspondingly, in the current study KDQOL-CF scores ≥74 were considered indicative of better cognitive function.
- ***Mentation: Center for Epidemiologic Studies***-***Depression (CES-D) scale.*** Depressive mood was assessed using the CES-D scale (20). Among dialysis patients, a CES-D score of 18 or higher is considered suggestive of clinical depression (21). Prescribed antidepressant medications identified in the medical chart, i.e. selective serotonin reuptake inhibitors (SSRIs), atypical antidepressants, and tricyclic antidepressants, provided an additional indicator.

### Analyses

Fried et al. (1) noted that there is limited information about the role of frailty in health outcomes for different subgroups, and this remains true with regard to older persons with dialysis-dependent CKD. Compared with characteristics of the overall in-center HD population in the U.S., the AAS enrolled a higher proportion of African-American and Asian persons, reflecting the population of the selected study sites (Atlanta GA and San Francisco CA metropolitan areas), and **Table 2** shows baseline characteristics of our study cohort by race/ethnicity categories. Summary statistics include the % women and % who had completed at least high school education; the median and range of participants’ age and time since kidney failure treatment start, i.e. vintage; and the distribution of baseline-assessed frailty status within race/ethnicity categories.

**Table 2:** Baseline characteristics of older persons with dialysis-dependent CKD who were eligible^a^ for this study, stratified by race and ethnicity^b^.

	African-American	White Non-Hispanic Hispanic		Asian
Number of persons	72	17	17	22
Age (years)				
Median	72	78	75	75
Observed range	65–87	67–83	66-89	65–88
**Women (%)**	61	6	59	45
^c^ESRD vintage (years)				
Median	3.3	2.0	3.4	3.3
Observed range	0.5–23.2	0.8—5.6	0.8—10.9	0.8—12.0
At least high school education (%)	25	100	53	82
Frailty status, baseline (n)				
Robust	21	6	2	4
Pre-frail	43	8	9	13
Frail	8	3	6	5

CKD, chronic kidney disease.

ESRD, end-stage renal disease.

Vintage, years from ESRD treatment start to study enrollment.

Frailty status: Robust, pre-frail, or frail based on Fried et al. (1) frailty phenotype.

a Eliglble: AAS participants age ≥65 at baseline who received maintenance HD treatment at one of the 14 outpatient dialysis study clinics from baseline through their 24-month follow-up, with frailty status assessments at baseline and 24 months. In addition to participants described above, data were obtained for 3 eligible Native Hawaiian/Pacific Islander (NHPI) participants: ages 68, 68 and 78; all women, vintage 2.5, 4.8, and 7.4 years; high school education 2 yes, 1 no; baseline frailty status 2 Pre-frail, 1 Frail. These participants are included in the data reported in **Tables 3**-**5**.

b Ethnicity: Only participants who self-identified as white race also identified themselves as Hispanic.

c ESRD: For consistency with the title of the AAS, we continue in this paper to reference “ESRD,” i.e. end-stage renal disease. The kidney disease community recently adopted a revised term, “ESKD,” i.e. end-stage kidney disease.

Participants’ frailty status at all assessments was classified as Robust, Pre-frail, or Frail based on the Fried et al. frailty index criteria: Robust=no criteria met; Pre-frail=1 or 2 criteria met; Frail=3 or more criteria met. Frailty status evolution can be classified as Stable (no change over time), Improvement, and Degradation, with subgroups within these categories (22). In this study, older AAS participants’ frailty status evolution as assessed at baseline (Time 1) and at 24-months (Time 2) was summarized and described by the following categories and subgroups:

(1) Stable Robust
(2) Improved
• Pre-frail to Robust
• Frail to Pre-frail
(3) Stable Pre-frail
(4) Worse or Stable Frail
• Robust to Pre-frail
• Pre-frail to Frail
• Robust to Frail
• Stable Frail

Finally, the % of participants in each frailty status evolution category who were non-sedentary based on ≥500 k/cal energy expenditure over all three timepoints was determined, using LTPA kcal/week values recorded for participants at baseline, 12-months, and 24-months. The % of participants in each frailty status evolution category who reported Vitality scores ≥55, PF scores ≥75, and CF scores ≥74 at their 24-month follow-up (indicators relevant to Age-friendly Health priorities) was also determined.

## Results

### Study population

**Table 2** displays baseline characteristics of the 131 participants who comprised the study cohort, stratified by race (African-American, white, Asian) and Hispanic ethnicity; characteristics of 3 Native Hawaiian/Pacific Islander (NHPI) older participants are also summarized. In the older AAS cohort that comprises our study population, the median age of African Americans was 72, and the median age of non-Hispanic whites was 78; the median age of both Hispanic whites and Asians was 75. Non-Hispanic whites had the lowest representation of women, the highest % of participants with at least a high school education, and the shortest average ESRD vintage. Within each race and ethnic group, a majority of participants were categorized at baseline as pre-frail.

### Evolution of frailty status among older persons with dialysis-dependent CKD

Frailty status of study participants at 24-months in relation to their frailty status at baseline is shown in **Table 3**. Improved frailty status included two groups: Pre-frail to Robust (n=11) and Frail to Pre-frail (n=13). Stable frailty status included Stable Robust (n=19), Stable Pre-frail (n=46), and Stable Frail (n=10). Finally, changers whose assessed status worsened from baseline to 24 months included three groups: Robust to Pre-frail (n=11); Robust to Frail (n=3); and Pre-frail to Frail (n=18).

**Table 3:** Frailty status of study participants at 24-months in relation to their frailty status at baseline.

	Participant Status at 24-month Follow-up			
Participant Status at Baseline	Robust	Pre-frail	Frail	Totals
Robust	19^b^	11^c^	3^c^	33
Pre-frail	11^a^	46^b^	18^c^	75
Frail	0	13^a^	10^b^	23
Totals	30	70	31	131

a Improved status, baseline to 24 months.

b Stable status, baseline to 24 months.

c Worse status, baseline to 24 months.

The purpose of **Table 4** is to summarize characteristics of the four groups of participants who were observed to maintain non-frail status, or to improve their frailty status, over the 24-month observation period. Among these four groups, the average age of participants was younger in the Stable Robust group, and women had lower representation in the Stable Robust group. Interestingly, there was little variation in the median ESRD vintage of participants who were classified as Stable Robust, Improved, and Stable Pre-frail.

**Table 4:** Participants with stable robust, improved, and stable pre-frail status as of their 24-month follow-up: Characteristics of older persons with dialysis-dependent CKD.

	Stable Robust	Improved Pre-frail → Robust	Improved Frail → Pre-frail	Stable Pre-frail
Number of persons	19	11	13	46
Age (years)				
Median	69	73	74	73
Observed range	65-83	66-80	65-85	66-88
**Women (%)**	37	54	46	56
ESRD vintage (yrs)				
Median	2.7	2.4	3.3	3.0
Observed range	1.0-9.4	0.8-5.3	0.8-13.5	0.7-14.8
Race/ethnicity (n)				
African-American	13	8	5	26
**White**	4 (3 non-Hispanic, 1 Hispanic)	2 (1 non-Hispanic, 1 Hispanic)	3 (Hispanic)	12 (5 non-Hispanic, 7 Hispanic)
Asian	2	1	4	6
NHPI			1	2

CKD, chronic kidney disease.

ESRD, end-stage renal disease.

Vintage, years from ESRD treatment start to study enrollment.

NHPI, Native Hawaiian/Pacific Islander.

### Non-sedentary behavior over the 24-month study period

Non-sedentary behavior provides a more dynamic view of individuals’ activity level over time compared with the dichotomous kcal/week metric for low activity level in the Fried et al. algorithm. Within frailty evolution categories (StabIe Robust, Improved, Stable Pre-frail, and Worse or Stable Frail), **Table 5** reports the proportion of participants who were non-sedentary based on ≥500 k/cal energy expenditure at all timepoints (baseline, 12 months, and 24 months). More than half of participants in the Stable Robust group and in the two Improved groups were classified as non-sedentary by this measure, but only one-third or fewer participants were non-sedentary in either the Stable Pre-frail group or the Worse groups. No participants in the Stable Frail group were classified as non-sedentary.

**Table 5:** Non-sedentary behavior over 24 months and health status scores at 24-months: Percentage of older persons with dialysis-dependent CKD, stratified by longitudinal frailty status.

Longitudinal frailty status	n	Non-sedentary behavior pattern	Health Status Scores at 24 m ^a^Vitality ≥55 ^a^PF ≥75 ^b^CF ≥74
**Stable Robust**	19	58%	89% 42% 95%
Improved			
Pre-frail to Robust	11	54%	64% 27% 73%
Frail to Pre-frail	13	73%	62% 8% 69%
**Stable Pre-Frail**	46	28%	56% 9% 78%
Worse			
Robust to Pre-frail	11	36%	64% 18% 82%
Robust (3)/Pre-frail (18) to Frail	21	35%	57% 10% 57%
**Stable Frail**	10	0	60% 0 50%

Non-sedentary behavior pattern, ≥500 kilocalories/week expended in Leisure Time Physical Activity reported over 24 months; CKD, chronic kidney disease; m, months; PF, Physical Function; CF, Cognitive Function

a SF-36, Medical Outcomes Study Short Form-36

b KDQOL, Kidney Disease Quality of Life

### Indicators of age-friendly health system priorities

**Table 5** also displays the proportion of participants in each frailty evolution group who reported SF-36 Vitality scores ≥55, PF scores ≥75, and CF scores ≥ 74 at their 24-month assessment. These health status measures provide indicators relevant for three Age Friendly Health priorities in the 4Ms framework–What Matters, Mobility, and Mentation. The designated cutoff values indicate better self-reported vitality, better physical function and better cognitive function (17, 19. The proportion of participants with scores above the cutoffs on these three indicators was higher in the Stable Robust group than in any other group, although half or more of the participants in all groups scored above the cutoffs on the Vitality scale and the Cognitive Function scale. Self-reported PF scores were generally low, however, with fewer than half of the participants in each group scoring ≥75 on this measure.

Mentation includes mood as well as cognitive function. Depressed mood often characterizes persons with CKD who require chronic dialysis, with prescription of antidepressants being common. For the full AAS cohort age ≥18, we found in a multivariable analysis adjusted for baseline frailty status that participants with a CES-D score 18+ (21), and/or prescribed antidepressants, had increased odds for participant-reported falls over the past 12 months [OR 1.83, 95% CI 1.23-2.74, *p*=0.003] compared with participants whose CES-D score was <18 and who had no prescribed antidepressants (23). In the current study that is limited to older AAS participants age ≥65, the proportion of individuals with an elevated CES-D score at 24-months and/or with antidepressant medication(s) prescribed in their medical chart, by frailty evolution category, was 10% for Stable Robust,14% for Improved, 21% for Stable Pre-frail, and 28% for participants classified as Worse or Stable Frail.

## Discussion

The goal of this study was to examine the spectrum of frailty dynamics over time in a cohort of older persons with dialysis-dependent CKD. There is extensive evidence showing individuals’ increased risk of experiencing functional loss and incurring adverse events in association with frailty, among older community-living persons (1) and among persons with dialysis-dependent CKD (8, 23–26). However, not all older people develop frailty, and more understanding is needed of possible “protective factors against frailty” (27).

The broad distribution of frailty status evolution (improved/worse/stable) among older persons with dialysis-dependent CKD in our study cohort is consistent with distributions reported for studies of community-living older persons. Over a 24-month study period 18% of older AAS participants improved, while 24% worsened, their frailty status, and the most common pattern was for individuals’ assessed frailty status to be the same at follow-up as at baseline. A recent review and meta-analysis of 16 studies (data for ~43,000 persons aged 60+) that reported transitions between frailty states among community-living older persons over a mean of 3.9 years indicated that approximately 10% of individuals improved while approximately 40% worsened their frailty status, and half or more of the older persons studied remained in the same frailty status (28). Gill et al. (29), who studied transitions between frailty states of 754 community-living persons aged ≥70 who were assessed for frailty at 18-month intervals over a 54-month period, found (a) that transition to a worse frailty state was more common than improvement during the 18-month intervals and (b) that it was common for individuals to remain in the same frailty state, especially in the first 18-month interval. To complement research that explores the *biological underpinnings of frailty*, Gill et al. (29), p.423) called for continued longitudinal research to investigate the *epidemiology of frailty* “including its natural course, risk factors, precipitants, and interrelationships with disability and comorbidity.”

Evidence for a pattern of non-sedentary behavior over time, estimated by kcal/week of LTPA, more often characterized participants in the Stable Robust and Improved status groups in our study, compared with participants categorized as Stable Pre-frail or Worse (**Table 5**). Cross-sectional and longitudinal studies in the general population have shown that a physically active lifestyle may have anti-inflammatory properties, but evidence from studies conducted with maintenance HD patients is limited. In a previously reported multivariable analysis using data for the full AAS cohort age ≥18, adjusted for participant age, we found that non-sedentary behavior was associated with lower log-normalized CRP over 24 months (13).

Within frailty evolution groups, we also report the proportion of participants who had higher scores on Vitality, PF and CF self-report scales. Vitality, PF, and CF were assessed by health status measures that are well validated in research with persons who have kidney disease. These health status domains are relevant for three priorities of the “4Ms” Framework of the Age Friendly Health System: *What Matters*, *Mobility*, and *Mentation*:

*What Matters*. Geriatric health care seeks to prioritize addressing “what matters” to individuals. While clinical trials with persons on HD typically focus on the outcomes of mortality, adverse events, and biological markers, patients tend to prioritize outcomes that are more relevant to their daily living and well-being. Persons with kidney disease, especially persons dependent on maintenance HD, have ranked fatigue/energy as a top priority outcome for clinical research, even ranking this outcome above survival (14).

*Mobility*. In the frailty index developed by Fried et al. (**Table 1**), mobility is assessed by a performance-based measure, walk speed. Self-rated physical function as measured by the SF-36 PF scale reflects individuals’ view of their ability to perform daily activities such as stair climbing and walking one or more blocks. Individuals may report more mobility limitation *via* the SF-36 PF self-report measure than would be expected based on their performance-based mobility evaluation (3). This difference might reflect, for example, a person’s experience of indoor or outdoor living environment challenges to mobility, which are not encountered during a performance evaluation of walk speed that is conducted in a well-lighted, even-surface indoor walkway. Thus, SF-36 PF scores are likely to reflect person-specific experiential factors as well as physical performance capability.

*Mentation*. Evidence of cognitive impairment and depressed mood is commonly observed among persons with dialysis-dependent CKD. In this study, the group defined as Stable Robust at 24 months had the largest proportion of individuals with high scores on the KDQOL-CF measure, as well as the lowest proportion of individuals with a CES-D score indicative of clinical depression and/or prescribed antidepressants.

It is true that many variables not discussed in this paper may be associated with patient-reported health status domains. The objective of this paper is to examine the association of frailty status over time with patient-reported health status, rather than to provide an in-depth examination of multiple variables associated with patient-reported health status. However, we suggest that frailty status serves to “capture” influence of other variables. In our prior work investigating frailty status of AAS participants, we have investigated the potential association of multiple variables with frailty status in this dataset (7). We refer interested readers to this work, in which univariable associations with frailty were observed for diabetes, cardiovascular conditions, peripheral vascular disease (PVD), serum albumin, and serum bicarbonate (but not for hemoglobin or Kt/V). In multivariable adjusted analyses, only “other cardiac diseases” (a category including cardiac dysrhythmia, atrial fibrillation, tachycardia, pericarditis, cardiac arrest, and transient ischemic attack), PVD, and serum albumin remained statistically significant risk factors associated with frailty. Of note, as we have reported (7), no significant interaction between participant age and any of these frailty predictor variables was observed in separate models that included (a) <65 vs. >65 years old and (b) age as a continuous variable.

A fourth priority focus of the Age Friendly Health System is *Medication*, which has recently been addressed in AAS data by Kimura et al. (30) for a subgroup of 337 younger and older participants. Acknowledging that both polypharmacy and frailty are highly prevalent among patients on HD and are associated with adverse outcomes, the authors examined longitudinal frailty status and the number of prescribed medications. The mean number of medications was 10 ± 5. Patients taking >11 medications showed higher odds for frailty at baseline than patients taking fewer than 8 medications (OR 1.54, 95% CI 1.05–2.26), and the incidence of frailty at 24 months was higher in those taking >11 medications (sub-distribution hazard ratio 2.15, 95% CI 1.32–3.48). Medication burden reflects disease burden among older persons with dialysis-dependent CKD, but there is growing recognition of polypharmacy issues and discussion of possible opportunities for deprescribing (e.g. 31).

Strengths of our research include careful assessment of frailty parameters in a longitudinal cohort design. In reporting our findings we relied on descriptive statistics appropriate for the small n’s in many groups and the categorical nature of assessed frailty status, which is best understood as indicative of a complex syndrome (1, 2). Our study focused on older dialysis survivors, and we acknowledge that, in addition to survivor bias, responses to some health status interview questions may have been influenced by social desirability bias on the part of participants.

We examined participant-reported scores on measures of perceived Vitality, physical function, and cognitive function that are relevant to three priorities of the Age-Friendly Health System–What Matters, Mobility, and Mentation. Compared with participants in other categories, a higher proportion of participants categorized as Stable Robust reported scores indicative of perceived Vitality, better physical function, and better cognitive function. At the same time, more than half of the participants in *all* frailty evolution categories reported scores indicating perceived Vitality and better cognitive function, which may be related to participants’ HD experience. Both fatigue complaint and cognitive impairment in persons undergoing HD are understood to have multiple potential etiologies, including the degree to which removal of uremic toxins and effective control of metabolic and fluid imbalance is accomplished *via* the dialysis procedure (32–34).

Participants’ perceived physical function scores indicated that Mobility was problematic for study participants. Fewer than half (49%) of participants categorized as Stable Robust reported better physical function, and the proportions were even lower among participants in the other categories. A key clinical implication of this study is the value of encouraging physical activity among persons with dialysis-dependent CKD, including older persons (3, 35). The significance that exercise may have for the aging process is well documented. Lifelong high-volume exercise training has been shown to increase mitochondrial volume and oxidative capacity, likely contributing to healthy aging (36). A history of high-volume exercise training is not common among persons with dialysis-dependent CKD, but even small amounts of activity, as opposed to remaining sedentary, can have multifactorial health benefits in CKD, as well as in non-CKD, populations (37, 38).

A related implication for clinical practice is the potential value of referral and access to physical therapy (PT) services for older persons undergoing chronic dialysis. PT services, which are underutilized in the dialysis-dependent CKD population, could have important implications for individuals’ resilience (39). Most individuals who require chronic HD receive treatment in outpatient dialysis centers, which should facilitate opportunity for provider referral to PT services. PT goals are patient-specific and include maintenance as well as improvement of function. We identified 32 Atlanta AAS participants for whom receipt of outpatient PT services was verified in Medicare claims data, and we examined activity and physical performance measures that were completed for these individuals before and after they received PT that included therapeutic exercises (CPT code 97110) “to develop strength and endurance, range of motion and flexibility.” Participants who received these PT services had increases in reported LTPA, as well as increases in performance-based mobility, strength, and balance scores (40).

The worldwide prevalence of frailty is expected to increase dramatically with growth of the geriatric population (27), and the same pattern is projected for persons with dialysis-dependent CKD (8). The initiatives of the Age-Friendly Health System call attention to important priorities for older persons in all healthcare settings. Our study suggests that strategies to promote resiliency among older adults with dialysis-dependent CKD could be informed by attention not only to frailty status transition that indicates improved status over time, but perhaps especially by attention to older adults’ maintenance of (stable) robust status over time. Ideally, efforts to promote resiliency would address multiple dimensions, including, but not limited to, sleep quality and psychosocial reserve, as well as dimensions of physiological resilience, such as balance, that are relevant for geriatric care in general (2) as well as for older persons living with dialysis-dependent CKD. A coordinated focus on mechanisms of resilience as well as frailty is a promising initiative in research and care for all older persons.

## Data availability statement

The original contributions presented in the study are included in the article/supplementary materials. Further inquiries can be directed to the corresponding author.

## Ethics statement

The studies involving human participants were reviewed and approved by Emory University Institutional Review Board. The patients/participants provided their written informed consent to participate in this study.

## Author contributions

Research area and study design: NGK, RZ; data acquisition: NGK, RZ; data analysis/interpretation: NGK, RZ; supervision: NGK. Each author contributed important intellectual content during manuscript drafting or revision and accepts accountability for the overall work by ensuring that questions pertaining to the accuracy or integrity of any portion of the work are appropriately investigated and resolved. NGK takes responsibility that this study has been reported honestly, accurately, and transparently. The authors served as Co-Principal Investigator (NGK) and Data Manager/Analyst (RZ) for the AAS, as members of the USRDS Special Studies Center on Rehabilitation and Quality of Life. All authors contributed to the article and approved the submitted version.
